# Dynamical features of stimulus integration by interacting cortical columns

**DOI:** 10.1186/1471-2202-14-S1-P268

**Published:** 2013-07-08

**Authors:** Robin Cao, Jochen Braun, Maurizio Mattia

**Affiliations:** 1 Cognitive Biology, Center for Behavioral Brain Sciences, Magdeburg, Germany; 2 Department of Technologies and Health, Istituto Superiore di Sanità, Roma, Italy

## 

In multi-stable perception and notably binocular rivalry, transitions between alternative perceptual states occur over a wide range of time-scales [1], but the distribution of dominance periods exhibits peculiar and consistent properties. For example, the tight correlation between mean and variance of this distribution represents a scalar property and a form of Weber's law [2]. Although several neural mechanisms for Weber's law have been proposed [3,4], here we focus on an alternative framework that involves interactions within a finite number of neural assemblies (such as cortical columns) encoding a distributed neural representation of a given perceptual state [5,6]. In this framework, each neural assembly shows nonlinear attractor dynamics (i.e., a double-well energy landscape) and switches spontaneously between inactive and active states due to endogenous noise. Energy landscape and, consequently, switching rates of each assembly are modulated by its external visual input and also by recurrent input from other assemblies [1,7]. A transition between one perceptual state and another is modelled as a two-stage process: first, the successive activation of assemblies due to external visual input ("stochastic integration") and, second, the snowballing activation of additional assemblies due to recurrent interactions ("global attractor dynamics") [1,6]. Whereas every local activation reflects a gain of evidence in favour of the associated perceptual state, the high-dimensional dynamics towards a global attractor state reflects the crossing of the perceptual threshold.

We present our progress towards an analytic treatment of this modelling framework. After reducing the non-linear input dependence of local assemblies to escape rates (Kramer's escape problem), the stochastic integration of a population of assemblies becomes a well-known birth-death process (Ehrenfest process). From the master equation of a finite number of *independent *assemblies, we recursively obtain exact analytical expressions for all moments of the first-passage time (FPT) distribution (if the perceptual threshold is modelled as an absorbing barrier). A scalar property holds in two regimes: a low-threshold, drift-dominated regime in which the threshold is independent of input and the FPT distribution is less skewed, and a high-threshold, fluctuation-dominated regime in which the threshold depends on input and the FPT distribution is more skewed. Finally, we consider synaptic couplings between cortical columns resorting to a mean-field approximation. In this framework, network dynamics of bistable assemblies are captured replacing constant transition rates by state-dependent rates. For strong enough couplings, more than one global attractor may coexist, and stochastic integration can be viewed as a slow descent across a rough surface until perceptual threshold is crossed [1]. In a low-threshold limit, we derive a uniform shift (time-warp) of the FPT distribution, allowing us to generalize the analytic expressions found earlier for independent assemblies. We conclude that this modelling framework contributes to the development of a general theory for multi-modular networks of neurons.
